# Seq2Ref: a web server to facilitate functional interpretation

**DOI:** 10.1186/1471-2105-14-30

**Published:** 2013-01-28

**Authors:** Wenlin Li, Qian Cong, Lisa N Kinch, Nick V Grishin

**Affiliations:** 1 Howard Hughes Medical Institute, University of Texas Southwestern Medical Center, Dallas, Texas 75390-9050, USA; 2 Department of Biochemistry and Department of Biophysics, University of Texas Southwestern Medical Center, Dallas, Texas 75390-9050, USA

**Keywords:** Web server, Functional interpretation, Sequence homology, Reference protein, PubMed literature

## Abstract

**Background:**

The size of the protein sequence database has been exponentially increasing due to advances in genome sequencing. However, experimentally characterized proteins only constitute a small portion of the database, such that the majority of sequences have been annotated by computational approaches. Current automatic annotation pipelines inevitably introduce errors, making the annotations unreliable. Instead of such error-prone automatic annotations, functional interpretation should rely on annotations of ‘reference proteins’ that have been experimentally characterized or manually curated.

**Results:**

The Seq2Ref server uses BLAST to detect proteins homologous to a query sequence and identifies the reference proteins among them. Seq2Ref then reports publications with experimental characterizations of the identified reference proteins that might be relevant to the query. Furthermore, a plurality-based rating system is developed to evaluate the homologous relationships and rank the reference proteins by their relevance to the query.

**Conclusions:**

The reference proteins detected by our server will lend insight into proteins of unknown function and provide extensive information to develop in-depth understanding of uncharacterized proteins. Seq2Ref is available at: http://prodata.swmed.edu/seq2ref.

## Background

Due to the avalanche of protein sequences made available by high-throughput genome sequencing, complete manual annotation is unfeasible, leaving a large fraction of protein functions to be predicted by automatic functional annotation pipelines [1]. However, without experimental characterization, the quality of annotation is often questionable, owing to errors in automatic annotation transfer and lack of updates from new findings. In spite of recent advances in highly integrative functional prediction methods [2], a recent investigation [3] into the annotation quality of well-characterized enzyme families revealed that the average percentage of misannotation for the haloacid dehalogenase (HAD) superfamily in the three largest public databases, i.e. non-redundant (nr) [4], TrEMBL [5] and KEGG [6], is over 60%. The possible causes of such annotation errors include multi-domain problems [7], experimental data misinterpretations, threshold relativity problems, and paralog-ortholog misclassifications [8-12]. Moreover, the simplified descriptions recorded in protein sequence and protein family databases are usually inadequate for understanding the precise function of a protein [1].

Such errors and omissions make database annotations insufficient for complete functional interpretation of a protein. A more accurate source of annotations is the ‘reference proteins’ closely related to the protein of interest. We define ‘reference, proteins’ as proteins that have been experimentally studied, manually curated, and reported in the literature. Information about reference proteins is essential for accurate functional interpretation and experimental design. The cross-links between proteins, genes, and associated literature available from National Center for Biotechnology Information (NCBI) provide a basis for reference protein identification. However, it is not trivial to identify a good set of reference proteins and supporting literature because such reference proteins constitute only a small portion of protein databases. Additionally, many proteins linked to large-scale studies (such as genome sequencing) do not provide sufficient functional information.

We have developed a web server named Seq2Ref to assist the identification of applicable reference proteins. Seq2Ref employs BLAST [13] to perform homology searches and exploits crosslinks created by NCBI between proteins and literature to detect reference proteins. Homologs from the Protein Data Bank (PDB) [14] and Swiss-Prot (SP) [15] databases are detected as well, as these databases contain experimental data on 3D protein structures and manually curated annotation on sequence records, respectively. Moreover, we developed a plurality-based rating system integrating reciprocal BLAST and Multiple Sequence Comparisons (MSC) to rank the reference proteins. By retrieving homologous reference proteins, Seq2Ref can contribute to precisely inferring unknown protein function and developing detailed functional interpretation.

## Results and discussion

### Server interface

**Figure 1:**
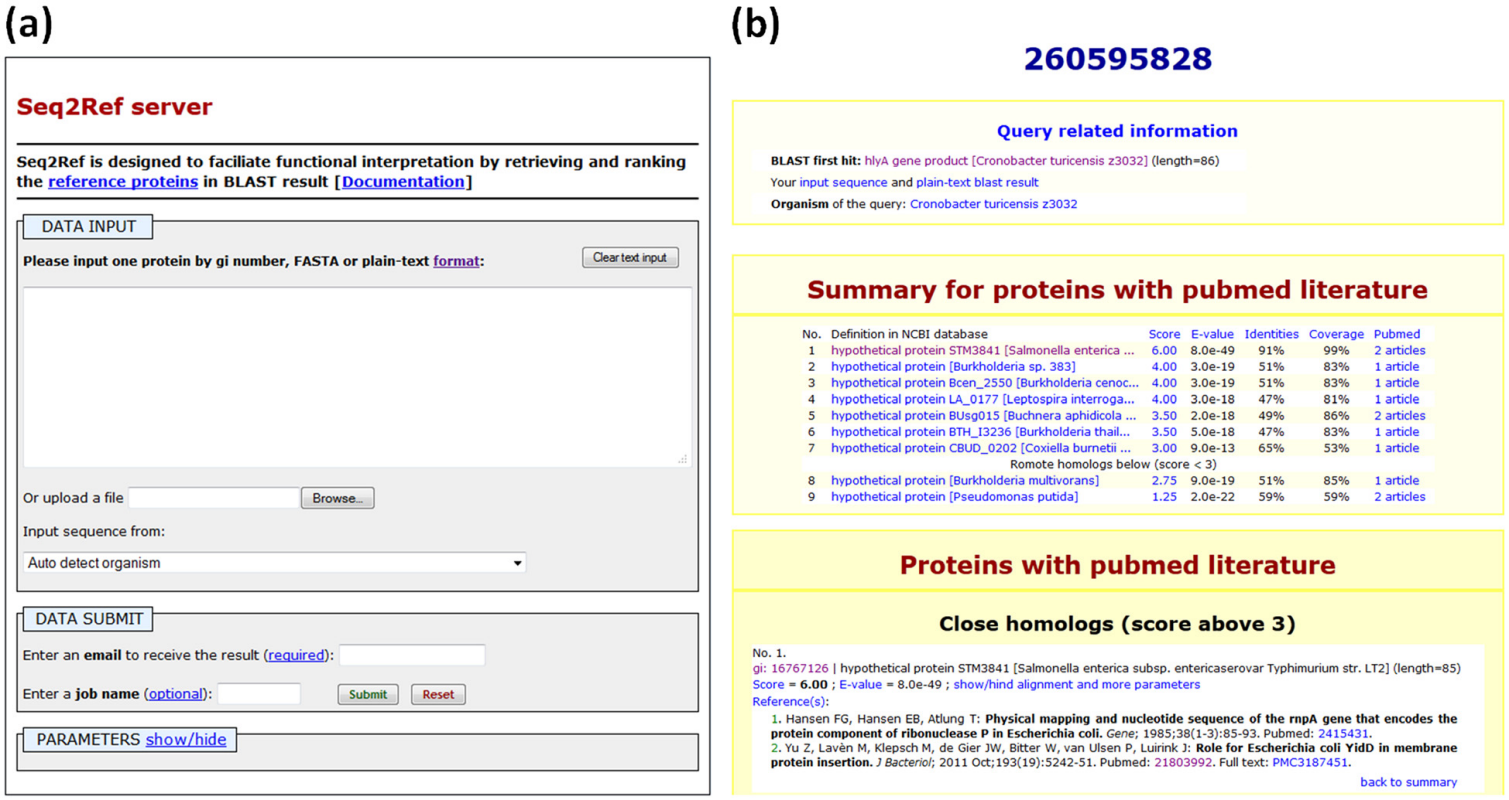
**The Seq2Ref webpage interfaces (a) A screenshot of the Seq2Ref submission interface shows the regions for the query input. **The query can be submitted by typing in the protein sequence or uploading a file containing the sequence in three formats (gene index (gi) number, FASTA or plain-text). An email address is required for receipt of results. (**b**) A screenshot shows the top region of the output interface. Query related information (top) is reported. Each section of the results contains a summary table and a detailed panel displaying reference proteins and their relevant information. Full result is in the link: http://prodata.swmed.edu/wenlin/server/user_data/seq2ref/S2RGsaCMG/result.html.

The input and output interfaces are shown in Figure 1. An email address and the query protein are the minimal requirements to initiate a job. Options for BLAST search parameters and selection of server modes (fast/slow) are available in the PARAMETERS panel. We recommend manually selecting the organism of the input sequence for reciprocal BLAST if the input sequence is not in the nr database. The total run time is usually 5 to 15 min for fast mode and 1 hour or more for slow mode. When the job completes, an email notification will be sent to the address provided by the user.

The results page (shown in Figure 1) lists the reference proteins and relevant information in a ranked order. Reference proteins from three sources are shown, respectively, as: (1) a summary table containing protein definition, rating score and BLAST statistics (expectation value, sequence identity and coverage); (2) and a detailed description panel with the rating records, BLAST statistics and scores, and relevant database information. Reference proteins are ranked first by the rating score and second by the expectation value; the publications associated with each protein are sorted by the publication date. As functional studies of remote homologs may not be applicable to the query protein, by default we do not display reference proteins with rating scores lower than 3 in the detailed description panel.

### Benchmark

To assess the performance of the Seq2Ref server, especially the ability of our plurality-based rating system to sort out the most relevant references, we applied our algorithm to the enolase superfamily, which has been thoroughly characterized and recorded in the Structure-Function Linkage Database (SFLD) [16-18]. The enolase superfamily contains seven subgroups, which are further divided into 20 families. Proteins within one family share the same substrate specificity and can be considered orthologs; proteins within one subgroup share the same general base(s) in the active site and have the similar catalytic mechanism [19]. For each family, we selected one representative sequence, usually the one with an available 3D structure (Additional file 1: Table S1), as the input for benchmark.

At each rating score cutoff, coverage and average accuracy were used as parameters to evaluate the performance of Seq2Ref (Table 1). The coverage is defined as the percentage of tested sequences that detect reference proteins above the score cutoff. The accuracy is defined as the average of the true positive rates among tested sequences above the score cutoff. Two criteria were used to define true positives: (1) in a stringent (family) context, a true positive hit must be from the same family as the query; and (2) in a broader (subgroup) context, hits from the same subgroup but from different families are also considered true. As shown in Table 1, the accuracy is always 100% with score cutoffs no less than 4; when the cutoff drops to 3, Seq2Ref reaches 100% coverage but starts to include those hits from the same subgroup but different families. Although the biased dataset from only one family might cause overfitting of the statistics, the benchmark suggests that accurate functional interpretation at family level should be achieved by utilizing the reference proteins with a score no less than 4. The information for marginal hits with scores between 3 and 4 is valuable to understand the broad function of the protein subgroup. However, one should not directly transfer the specific functions of marginal hits, such as substrate specificity, to the query.

**Table 1:** The accuracy and coverage of the rating system

**Score**	**Subgroup accuracy**^**1**^	**Family accuracy**^**1**^	**Coverage**^**2**^
6	100%	100%	80%
>=5	100%	100%	85%
>=4	100%	100%	95%
>=3	100%	78.5%	100%

1: accuracy calculated by averaging the family/subgroup true positive rates.

2: coverage calculated by taking the ratio of testing sequences that detect reference proteins above the score cutoff.

### Case study and examples

Due to its ability to retrieve reference proteins and their relevant information in a ranked order, the Seq2Ref server is useful for finding PubMed references relevant to proteins of unknown function, as well as obtaining a deeper understanding of proteins than that revealed by short annotations, as illustrated by the following examples.

#### Organizing new information for proteins of unknown function

Hypothetical proteins of unknown function constitute a remarkably large portion of the database [20]. Novel studies on uncharacterized proteins and their orthologs provide new insights about their functions, but sequence databases often do not incorporate this information in a timely manner. By retrieving literature, Seq2Ref helps to obtain the most recent information about proteins.

The *Macaca mulatta* protein, corresponding to gi|355567738, is annotated as a hypothetical protein EGK_07670 in the NCBI Protein database (Seq2Ref results: http://prodata.swmed.edu/wenlin/server/user_data/seq2ref/S2Rnv4cun/result.html). This hypothetical protein contains three conserved domains of unknown function (two DUF3730 and one DUF3028). Our server detects one close homolog, a hypothetical protein (gi|23345097) in human, which has been experimentally studied. The highly confident statistics in BLAST (e-value around 0; 98% identity, 100% coverage) and similar protein domain composition support an orthologous relationship between these two proteins. The human protein was recently (in 2012) reported to be a tumor suppressor in gliomas. It was named ‘focadhesin’, due to its cellular localization at the focal adhesion of the cell membrane [21]. As a likely ortholog, the *M. mulatta* protein might also be a tumor suppressor and localized at the focal adhesion. Thus, by finding a homolog with the latest experimental publication not yet incorporated in sequence databases, Seq2Ref can serve as a basis for reliable functional prediction of unknown proteins.

#### Providing detailed information about a protein’s function

Although conserved domains in proteins usually suggest their functions, overly broad descriptions of domain functions are less informative than more specific descriptions. By presenting reference proteins and associated literature, Seq2Ref can offer more definitive and reliable information about protein functions.

One example is the hlyA gene product in *Cronobacter turicensis z3032* (gi|260595828, Seq2Ref results: http://prodata.swmed.edu/wenlin/server/user_data/seq2ref/S2RGsaCMG/result.html). A search of the Conserved Domain Database (CDD) merely suggests that this protein contains a ‘haemolytic domain’, with the most similar hit (lowest expectation value) annotated as a ‘hypothetical protein’ and one possible informative hit as ‘conserved hypothetical protein YidD’. The ‘conserved hypothetical protein YidD’ domain (TIGR00278) shows neither functional studies nor a detailed functional description. The publication [22] associated with a Pfam domain record (pfam01809) in the CDD search result suggests that the name ‘haemolytic domain’ originated because one protein (ytjA from *Bacillus subtilis*) containing this domain can cause cells to lyse in culture. Unfortunately, this study failed to suggest a specific molecular function. Seq2Ref provided more information by detecting (e-value 8.0e-49; 90% identity; reciprocal best hit) the experimentally studied protein YidD from *E. coli* (gi|67476547), which is identical to the NCBI nr database representative protein (gi|16767126) from *Salmonella enterica*. This orthology is reinforced by the common conserved genomic context [23] (Additional file 2: Table S2) and the CLANS [24] protein similarity network, in which *E. coli* YidD and *C. turicensis* hlyA cluster tightly together among their homologs (Additional file 3: Figure S1). The reference [23] associated with *E. coli* YidD detected by our server suggests that YidD assists YidC, the protein insertase, in insertion of inner membrane proteins. As a confident ortholog of *E. coli* YidD, the hlyA gene from *C. turicensis* very likely shares the same function. Thus, the homologous reference protein, detected by Seq2Ref, contributes to understanding the protein function more specifically.

### Limitations

As shown in the examples, the Seq2Ref server detects reference proteins, which can facilitate deeper understanding of the protein function. However, we should keep in mind the limitations. The main concern regards the quality of cross-links between the NCBI Protein and PubMed databases. Missing or wrong links defined by NCBI would result in the loss of or the inappropriate assignment of relevant literature. Another concern is that although the top ranked reference proteins are very likely functionally similar to the query proteins, one should still be careful in directly transferring the information from the hit to the query, as verification of orthology requires additional diligent analysis. To come to the best conclusions about a protein’s function, one should critically inspect the relevance of the publications and the homology of the reference proteins to the query.

## Conclusions

Seq2Ref is a homology-based tool to identify reference proteins from PubMed, PDB and SP databases. We have developed a plurality-based rating system that evaluates homologous relationships to indicate the degree of confidence one should have in transferring annotations from a well-studied reference protein to a similar new protein. Thus, by retrieving both experimental studies and high-quality functional annotations of reference proteins, our server provides a solid basis for correct function interpretation of novel proteins.

## Methods

### Detection of homologs and identification of reference proteins

The workflow of the Seq2Ref server is available in Additional file 4: Figure S2. Seq2Ref performs the BLAST search against the NCBI nr database to detect homologs of the query protein. Based on BLAST search results, reference proteins are identified as: (1) the hits linked to PubMed literature by NCBI (those publications associated with more than 100 protein records are excluded); (2) the hits from PDB; (3) and the hits from SP. Reference proteins from PDB and SP databases are obtained by parsing the protein descriptions recorded in nr. Retrieval from PubMed requires fast but thorough searching of cross-links between NCBI databases. To implement this search, Seq2Ref has two modes: a “fast mode” based on searching a pre-processed local database (updated every 6 months) that consists of the reference proteins in nr, and a “slow mode” in which the most updated reference proteins are retrieved in real-time via NCBI Entrez [25].

### Analysis of homologous relationship

We assign orthology firstly by the approximate method of reciprocal best hits [26]. In this method, it is necessary to know the the source-organism of the query protein. To automatically detect the species, Seq2Ref identifies the taxon of the first BLAST hit with at least 97% identity and 90% coverage. Alternatively, the user can manually specify the organism of the input sequence. To avoid possible false negatives caused by variants of the same gene in reciprocal BLAST, such as alleles containing a single nucleotide polymorphism, we pre-cluster the proteins from each genome using CD-HIT [27] (identity cutoff: 97%; coverage cutoff: 90%).

**Figure 2:**
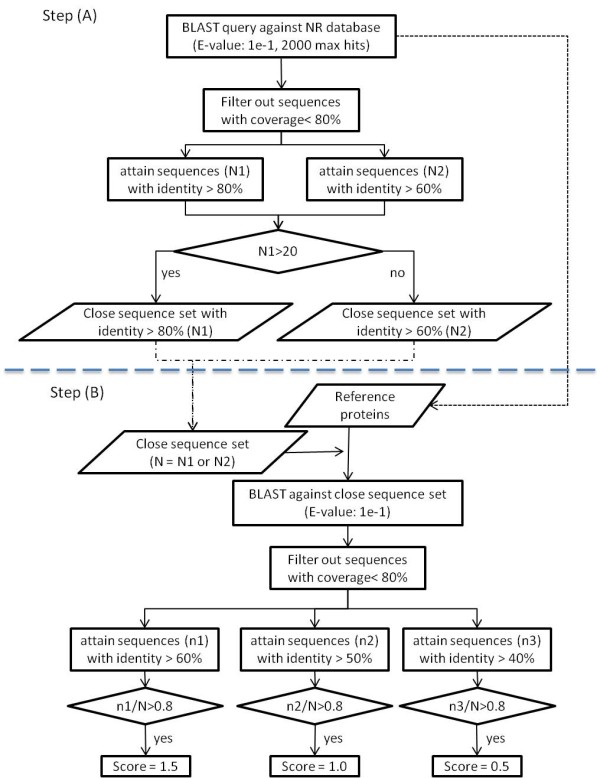
**Flow chart of multiple sequence comparison (MSC) method. **In step (**A**), MSC uses BLAST with filters as indicated to define a group of closely related sequences to the query (close sequence set, N); in Step (**B**), the Reference protein set consists of protein sequences identified without filters by BLAST in step A that are 1) from the Swiss-Prot database, 2) from the Protein Data Bank (PDB), and 3) linked to PubMed articles in the NCBI. Reference protein sequences are then used as queries against a database consisting of the close sequence set. Hits are filtered as indicated. If the filtered hit ratio is larger than 0.8, then a score is assigned to the reference protein.

Reference proteins are further analyzed by the method of multiple sequence comparison (MSC) shown in Figure 2. Specifically, we retrieve the sequences most closely related to the query, and then compare the reference proteins to those closely related sequences. Such multiple comparisons allow us to obtain more robust statistics in evaluating homology compared to simple pairwise comparison.

### Rank reference proteins by relevance to the query

We developed a plurality-based rating system with scores ranging from 1 to 6, with 6 indicating the most relevant hits (shown as Table 2). To note, our rating system aims to provide intuitive indicators for the level of similarity, but not act as a statistical predictor of functionality. Four features are considered: reciprocal BLAST, MSC, pairwise comparison between the query and the hit, and whether the hit protein is a “reference protein”, e.g. if there are PubMed citations linked to the protein in the current version of NCBI databases. The maximal rating score for each aspect is 2, 1.5, 1.5 and 1, respectively. A higher total rating score indicates the query protein is closer to the hit and is more likely to function similarly. Proteins with scores lower than 3 would be considered more distant homologs whose functions may have diverged, because they are neither reciprocal BLAST best hits nor with confident statistics in MSC and pairwise comparison.

**Table 2:** The rating system

**Feature**	**Criterion**	**Points**		
		**True**	**False**	**NA**
Reciprocal BLAST	Query-to-hit-genome^1^ best hit	+1	0	+0.25^3^
	Hit-to-query-genome^2^ best hit	+1	0	+0.25^3^
Multiple Sequence Comparison (MSC) Method^4^	Accept with identity cutoff 60%	+0.5	0	\
	Accept with identity cutoff 50%	+0.5	0	\
	Accept with identity cutoff 40%	+0.5	0	\
Pairwise comparison to the query^4^	Identity>60%	+0.5	0	\
	Identity>50%	+0.5	0	\
	Identity>40%	+0.5	0	\
others	Reference proteins	+1	0	\

1: use the input sequence to BLAST against the genome of the hit.

2: use the hit to BLAST against the genome of the input sequence.

3: the whole genome sequences of the protein are unavailable.

4: coverage in both the input sequence and the hits must larger than 80%.

## Competing interests

The authors declare that they have no competing interests.

## Authors’ contributions

WL carried out most of the work and drafted the manuscript. QC and LNK participated in the server design and helped draft the manuscript. NVG conceived the study, participated in its design, and helped draft the manuscript. All authors read and approved the final manuscript.

## Supplementary Material

Additional file 1: Table S1. The benchmark result links of enolase superfamily. The table shows the selected representative proteins for families in enolase superfamily and the webpage links of the server results. Click here for file

Additional file 2: Table S2. Pairwise BLAST results for proteins located in the operon containing YidD. Five proteins from *Escherichia coli *are compared with corresponding proteins in *Cronobacter turicensis.* Click here for file

Additional file 3: Figure S1. The protein similarity network of YidD homologs produced by CLANS program. Each black dot represents one protein sequence. Red circle and green asterisk represent the query (*Cronobacter turicensis* hlyA) protein and the experimental studied hit (*E. coli* YidD), respectively. Edges (lines) show BLAST connections between sequences that have an E-value at least as good as 10^−33^. Lengths of edges indicate that sequences in tightly clustered groups are relatively more similar to each other than sequences with few and distant connections. Click here for file

Additional file 4: Figure S2. The workflow of Seq2Ref. The whole process can be divided into homologous reference protein detection (Step 1) and homology evaluation (Step 2). Starting from a query sequence, a BLAST/PSI-BLAST search of the NCBI non-redundant database (NR) is performed (Step 1–1) to detect homologous proteins. Seq2Ref detects the reference protein among these homologous proteins by retrieving and checking the information in NCBI databases (Step 1–2). Orange lines represent the reference proteins among the BLAST result. Sequentially, Reciprocal BLAST (RB) and multiple sequence comparison (MSC) will be performed to evaluate the homologous relationships (Step 2–1). Integrating the statistics calculated above, a rating system will assign scores and rank the reference proteins (Step 2–2). Click here for file

## References

[B1] Valencia A Automatic annotation of protein function Curr Opin Struct Biol 2005 15 3 267 274 10.1016/j.sbi.2005.05.010 15922590

[B2] Rentzsch R Orengo CA Protein function prediction–the power of multiplicity Trends Biotechnol 2009 27 4 210 219 10.1016/j.tibtech.2009.01.002 19251332

[B3] Schnoes AM Brown SD Dodevski I Babbitt PC Annotation error in public databases: misannotation of molecular function in enzyme superfamilies PLoS Comput Biol 2009 5 12 e1000605 10.1371/journal.pcbi.1000605 20011109 PMC2781113

[B4] Benson DA Karsch-Mizrachi I Lipman DJ Ostell J Sayers EW GenBank Nucleic Acids Res 2010 38 Database issue D46 51 19910366 10.1093/nar/gkp1024 PMC2808980

[B5] The Universal Protein Resource (UniProt) in 2010 Nucleic Acids Res 2010 38 Database issue D142 148 2808944 19843607 10.1093/nar/gkp846 PMC2808944

[B6] Kanehisa M Araki M Goto S Hattori M Hirakawa M Itoh M Katayama T Kawashima S Okuda S Tokimatsu T KEGG for linking genomes to life and the environment Nucleic Acids Res 2008 36 Database issue D480 484 18077471 10.1093/nar/gkm882 PMC2238879

[B7] Kim BH Cong Q Grishin NV HangOut: generating clean PSI-BLAST profiles for domains with long insertions Bioinformatics 2010 26 12 1564 1565 10.1093/bioinformatics/btq208 20413635 PMC2881392

[B8] Galperin MY Koonin EV Sources of systematic error in functional annotation of genomes: domain rearrangement, non-orthologous gene displacement and operon disruption In Silico Biol 1998 1 1 55 67 11471243

[B9] Sasson O Kaplan N Linial M Functional annotation prediction: all for one and one for all Protein science: a publication of the Protein Society 2006 15 6 1557 1562 10.1110/ps.062185706 16672244 PMC2242553

[B10] Bork P Bairoch A Go hunting in sequence databases but watch out for the traps Trends in genetics: TIG 1996 12 10 425 427 10.1016/0168-9525(96)60040-7 8909140

[B11] Doerks T Bairoch A Bork P Protein annotation: detective work for function prediction Trends in genetics: TIG 1998 14 6 248 250 10.1016/S0168-9525(98)01486-3 9635409

[B12] Smith TF Zhang X The challenges of genome sequence annotation or “the devil is in the details” Nat Biotechnol 1997 15 12 1222 1223 10.1038/nbt1197-1222 9359093

[B13] Altschul SF Madden TL Schaffer AA Zhang J Zhang Z Miller W Lipman DJ Gapped BLAST and PSI-BLAST: a new generation of protein database search programs Nucleic Acids Res 1997 25 17 3389 3402 10.1093/nar/25.17.3389 9254694 PMC146917

[B14] Berman HM Westbrook J Feng Z Gilliland G Bhat TN Weissig H Shindyalov IN Bourne PE The protein data bank Nucleic Acids Res 2000 28 1 235 242 10.1093/nar/28.1.235 10592235 PMC102472

[B15] Bairoch A Boeckmann B Ferro S Gasteiger E Swiss-Prot: juggling between evolution and stability Brief Bioinform 2004 5 1 39 55 10.1093/bib/5.1.39 15153305

[B16] Brown SD Gerlt JA Seffernick JL Babbitt PC A gold standard set of mechanistically diverse enzyme superfamilies Genome Biol 2006 7 1 R8 10.1186/gb-2006-7-1-r8 16507141 PMC1431709

[B17] Pegg SC Brown S Ojha S Huang CC Ferrin TE Babbitt PC Representing structure-function relationships in mechanistically diverse enzyme superfamilies Pacific Symposium on Biocomputing Pacific Symposium on Biocomputing 2005 358 369 15759641

[B18] Pegg SC Brown SD Ojha S Seffernick J Meng EC Morris JH Chang PJ Huang CC Ferrin TE Babbitt PC Leveraging enzyme structure-function relationships for functional inference and experimental design: the structure-function linkage database Biochemistry 2006 45 8 2545 2555 10.1021/bi052101l 16489747

[B19] Babbitt PC Hasson MS Wedekind JE Palmer DR Barrett WC Reed GH Rayment I Ringe D Kenyon GL Gerlt JA The enolase superfamily: a general strategy for enzyme-catalyzed abstraction of the alpha-protons of carboxylic acids Biochemistry 1996 35 51 16489 16501 10.1021/bi9616413 8987982

[B20] Jaroszewski L Li Z Krishna SS Bakolitsa C Wooley J Deacon AM Wilson IA Godzik A Exploration of uncharted regions of the protein universe PLoS Biol 2009 7 9 e1000205 10.1371/journal.pbio.1000205 19787035 PMC2744874

[B21] Brockschmidt A Trost D Peterziel H Zimmermann K Ehrler M Grassmann H Pfenning PN Waha A Wohlleber D Brockschmidt FF KIAA1797/FOCAD encodes a novel focal adhesion protein with tumour suppressor function in gliomas Brain: a journal of neurology 2012 135 Pt 4 1027 1041 22427331 10.1093/brain/aws045

[B22] Liu J Fang C Jiang Y Yan R Characterization of a hemolysin gene ytjA from Bacillus subtilis Curr Microbiol 2009 58 6 642 647 10.1007/s00284-009-9383-1 19306042

[B23] Yu Z Laven M Klepsch M de Gier JW Bitter W van Ulsen P Luirink J Role for escherichia coli YidD in membrane protein insertion J Bacteriol 2011 193 19 5242 5251 10.1128/JB.05429-11 21803992 PMC3187451

[B24] Frickey T Lupas A CLANS: a Java application for visualizing protein families based on pairwise similarity Bioinformatics 2004 20 18 3702 3704 10.1093/bioinformatics/bth444 15284097

[B25] Sayers EW Barrett T Benson DA Bolton E Bryant SH Canese K Chetvernin V Church DM Dicuccio M Federhen S Database resources of the national center for biotechnology information Nucleic Acids Res 2012 40 Database issue D13 25 22140104 10.1093/nar/gkr1184 PMC3245031

[B26] Moreno-Hagelsieb G Latimer K Choosing BLAST options for better detection of orthologs as reciprocal best hits Bioinformatics 2008 24 3 319 324 10.1093/bioinformatics/btm585 18042555

[B27] Li W Godzik A Cd-hit: a fast program for clustering and comparing large sets of protein or nucleotide sequences Bioinformatics 2006 22 13 1658 1659 10.1093/bioinformatics/btl158 16731699

