# Supplementary material for: Seq2Ref: a web server to facilitate functional interpretation
Source: BMC Bioinformatics. 2013 Jan 28;14:30. doi: 10.1186/1471-2105-14-30 (PMC3573977; doi:10.1186/1471-2105-14-30)

Figure S1. **The protein similarity network of YidD homologs produced by CLANS program.**

Each black dot represents one protein sequence. Red circle and green asterisk represent the query (*Cronobacter turicensis* hlyA) protein and the experimental studied hit (*E. coli* YidD), respectively. Edges (lines) show BLAST connections between sequences that have an E-value at least as good as 10^−33^. Lengths of edges indicate that sequences in tightly clustered groups are relatively more similar to each other than sequences with few and distant connections.


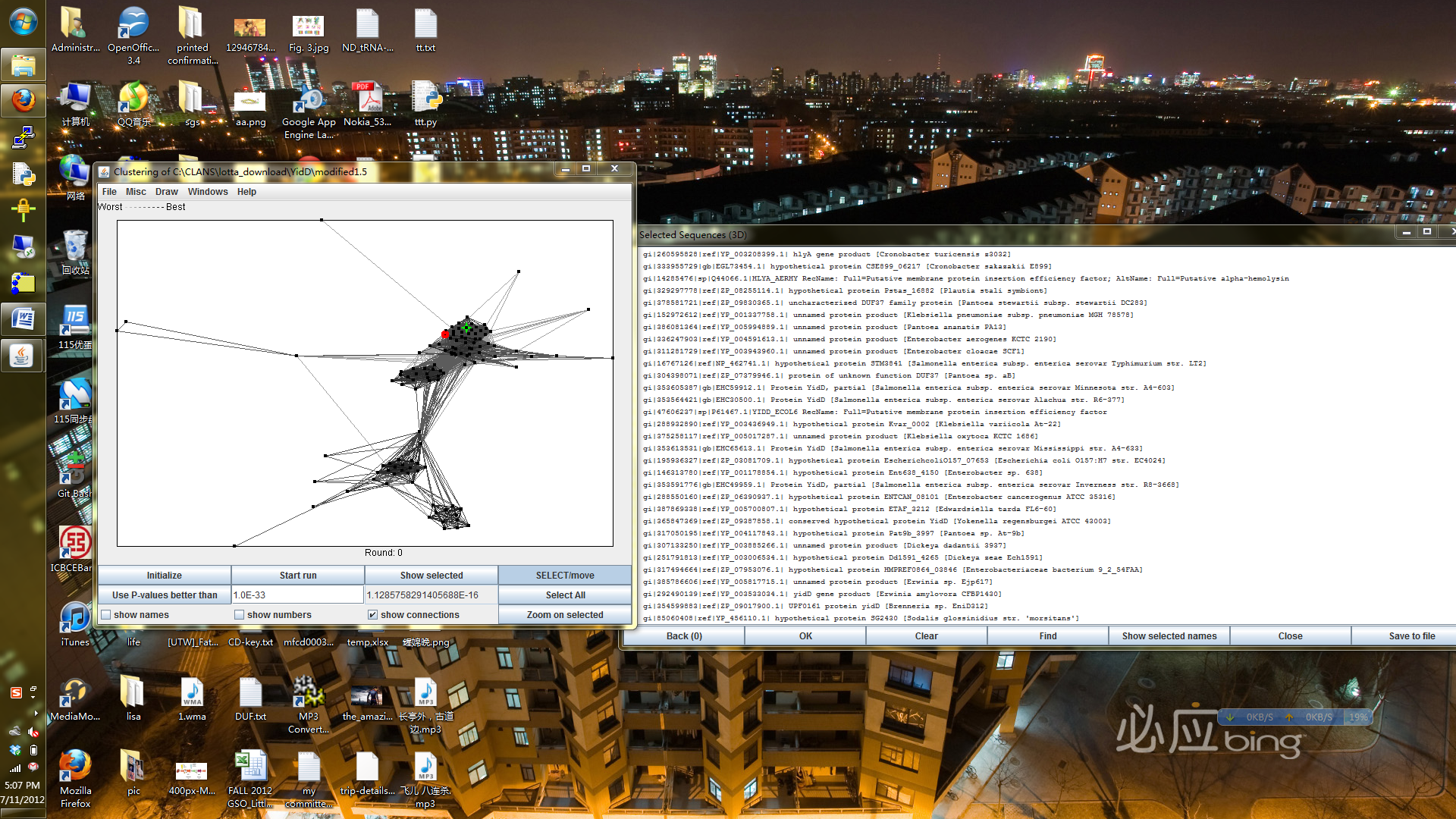

Supplement: Additional file 3: Figure S1. — The protein similarity network of YidD homologs produced by CLANS program. Each black dot represents one protein sequence. Red circle and green asterisk represent the query (Cronobacter turicensis hlyA) protein and the experimental studied hit (E. coli YidD), respectively. Edges (lines) show BLAST connections between sequences that have an E-value at least as good as 10−33. Lengths of edges indicate that sequences in tightly clustered groups are relatively more similar to each other than sequences with few and distant connections. [file 1471-2105-14-30-S3.docx]
