# Supplementary material for: Seq2Ref: a web server to facilitate functional interpretation
Source: BMC Bioinformatics. 2013 Jan 28;14:30. doi: 10.1186/1471-2105-14-30 (PMC3573977; doi:10.1186/1471-2105-14-30)
Supplement: Additional file 4: Figure S2. — The workflow of Seq2Ref. The whole process can be divided into homologous reference protein detection (Step 1) and homology evaluation (Step 2). Starting from a query sequence, a BLAST/PSI-BLAST search of the NCBI non-redundant database (NR) is performed (Step 1–1) to detect homologous proteins. Seq2Ref detects the reference protein among these homologous proteins by retrieving and checking the information in NCBI databases (Step 1–2). Orange lines represent the reference proteins among the BLAST result. Sequentially, Reciprocal BLAST (RB) and multiple sequence comparison (MSC) will be performed to evaluate the homologous relationships (Step 2–1). Integrating the statistics calculated above, a rating system will assign scores and rank the reference proteins (Step 2–2). [file 1471-2105-14-30-S4.docx]

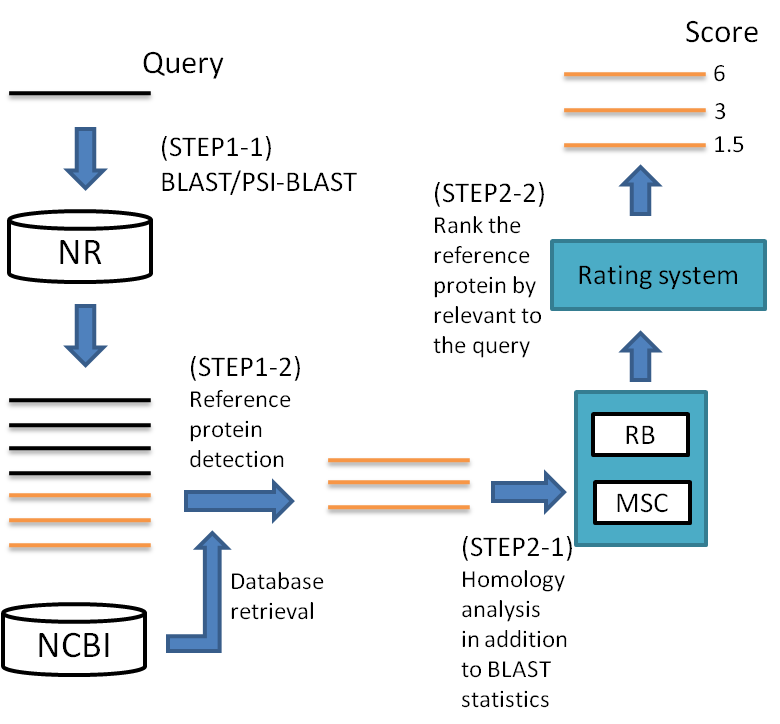


Figure S2. the workflow of Seq2Ref. The whole process can be divided into homologous reference protein detection (Step 1) and homology evaluation (Step 2). Starting from a query sequence, a BLAST/PSI-BLAST search of the NCBI non-redundant database (NR) is performed (Step 1-1) to detect homologous proteins. Seq2Ref detects the reference protein among these homologous proteins by retrieving and checking the information in NCBI databases (Step 1-2). Orange lines represent the reference proteins among the BLAST result. Sequentially, Reciprocal BLAST (RB) and multiple sequence comparison (MSC) will be performed to evaluate the homologous relationships (Step 2-1). Integrating the statistics calculated above, a rating system will assign scores and rank the reference proteins (Step 2-2).
